# Ileocaecal Tuberculosis Presenting with Massive Hematochezia: A Rare Clinical Manifestation

**DOI:** 10.1155/2021/1161135

**Published:** 2021-12-10

**Authors:** Neha Mehta, Man Bahadur Paudyal, Sangam Shah, Rajan Chamlagain, Moon Shrestha, Ashish Mehta, Prabin Bhattarai

**Affiliations:** ^1^Tribhuvan University Teaching Hospital, Maharajgunj 44600, Nepal; ^2^Maharajgunj Medical Campus, Institute of Medicine, Tribhuvan University, Maharajgunj 44600, Nepal; ^3^BP Koirala Institute of Health Sciences, Dharan 56700, Nepal; ^4^Department of Internal Medicine, Maharajgunj Medical Campus, Institute of Medicine, Tribhuvan University, Maharajgunj 44600, Nepal

## Abstract

Abdominal pain, diarrhea, weight loss, anorexia, and fever are common symptoms of intestinal tuberculosis, while bleeding from the lumen, intestinal obstruction, perforation, and fistula formation are uncommon presentations in ileocaecal tuberculosis. Here, we present a case of a 33-year-old male with intestinal tuberculosis who initially presented with massive bleeding per rectum.

## 1. Introduction

Tuberculosis is a public health issue in Nepal which is the leading cause of deaths. In Nepal, the incidence of tuberculosis in 2019/20 is 151 per 100,000, with 29% being extrapulmonary [1]. Gastrointestinal tuberculosis is a less-common manifestation of extrapulmonary tuberculosis [2]. Due to overlapping clinical, radiological, and endoscopic characteristics, abdominal tuberculosis is frequently misdiagnosed as Crohn's disease, colonic cancer, and abdominal lymphoma [3]. The signs of abdominal tuberculosis are obscure, creating a delay in diagnosis and treatment, resulting in severe morbidities. The clinical manifestations of abdominal TB differ depending on the site of infection. Abdominal pain, diarrhea, weight loss, anorexia, and fever are common symptoms of intestinal tuberculosis, although bleeding from the lumen, intestinal obstruction, perforation, and fistula formation are uncommon [2, 4]. Here, we present a case of a 38-year-old male with intestinal tuberculosis who initially presented with massive bleeding per rectum.

## 2. Case Presentation

A 33-year-old male presented to our center with chief complaints of profuse per rectal bleed mixed with stool for three days that was associated with easy fatigability for one week prior to the initial presentation. He also had one episode of black tarry stool. However, he had no complaints of blood in vomit, purpuric rashes, or petechiae. He also had no hematuria, weight loss, night sweats, evening rise of temperature or loss of appetite, cough, chest pain, dyspnea, palpitation, limb edema, loose stools, jaundice, and abdominal distension. Bleeding was absent from other orifices. He had no history of diabetes mellitus, hypertension, cardiac diseases, and pulmonary tuberculosis in the past. He consumed 80 grams of alcohol per day for 15 years, but he did not smoke.

On examination, he was ill looking, conscious, and was well oriented to time place and person. He had pallor and was dehydrated. However, he had no icterus, clubbing, cyanosis, or edema. His pulse rate was 110 beats/minute, blood pressure was 80/60 mm of Hg, body temperature was 98°F (36.6°C), respiratory rate was 19 breaths/minute, and oxygen saturation was 95% in room air. The digital rectal examination showed fresh blood over the examining finger and otherwise normal findings. Abdominal and cardiac examination was normal.

Laboratory investigations showed hemoglobin 10.8 g/dl and hematocrit 31.6%. The total leukocyte count was 11510/mm^3^, neutrophils were 78%, and platelet count was 291000/mm^3^. The prothrombin time was 14 seconds, and the International normalized ratio was 1.08. The albumin level in the blood was 2.4 gm/dl, and total protein was 6.1 gm/dl, total and direct bilirubin were 0.7 and 0.1 mg/dl in the blood. Alanine aminotransferase and aspartate aminotransferase level was 97 U/L and 114 U/L, respectively. HbA_1_C level in the blood was 7.2. The level of urea (33 mg/dl), creatinine (1.1 mg/dl), sodium (132 mEq/l), and potassium (3.6 mEq/l) were within normal range. Traces of sugar and albumin (++) were present in the urine examination. The fecal occult blood was found in the stool. *Mycobacterium tuberculosis* was not detected in sputum in the acid-fast bacilli (AFB) stain.

**Figure 1:**
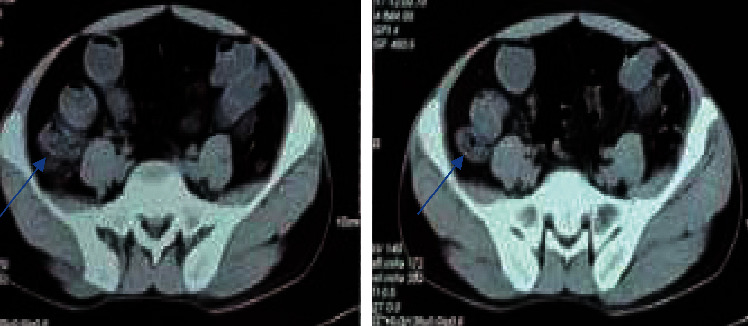
CT of the abdomen showing asymmetric circumferential thickening in the ileocaecal region with lobulated thickened caecum, soft-tissue stranding, and necrotic mesenteric lymphadenopathy.

The chest X-ray was normal. Ultrasonography (USG) of the abdomen and pelvis and upper gastrointestinal endoscopy showed normal findings. A Computed Tomography (CT) scan of chest and abdomen showed asymmetric circumferential thickening in the ileocaecal region with lobulated thickened caecum, soft-tissue stranding and necrotic mesenteric lymphadenopathy, and indeterminate lobule in the lung (Figure 1). Colonoscopy showed multiple transverse ulcers with overlying exudates in terminal ileum and ascending and transverse colon. Histopathological examination of the ileum and colon showed patchy ulcers with exudates, granulation tissue, fibrosis, deep lymphoplasmocytic inflammation, and crypt regenerative changes with fibrinoid changes in scattered capillaries and venules and was inconclusive. However, gene XPERT was positive for *Mycobacterium tuberculosis*.

He was diagnosed with ileocaecal tuberculosis. The hemoglobin level dropped to 6.1 g/dl after two days of admission, and he had an episode of weakness associated with profuse sweating and rigor. Fluid resuscitation followed by two pints of whole blood transfusion was performed. After that, his hemoglobin was increased to 10.2 mg/dl. He was managed with four antitubercular drugs and showed significant improvement. Following this, he was discharged on oral antitubercular medications (isoniazid, rifampicin, pyrazinamide, ethambutol, and pyridoxine) after 10 days of admission. On follow-up after two weeks, he was responding well to antitubercular medications with improved symptoms, i.e., no per rectal bleeding, and his liver function test was within the normal range.

## 3. Discussion

The ileocaecal region is the most usually affected by gastrointestinal TB, accounting for 64%, followed by the jejunum and large intestine. The ileocaecal region is the most involved site in the gastrointestinal tract because it has a prolonged fecal stasis, a high density of lymphoid tissue, a neutral pH environment, and absorptive transport mechanisms that allow ingested mycobacterium to be absorbed [5]. Ileocaecal TB is difficult to diagnose since it resembles other conditions such as Crohn's disease, amebiasis, diverticulitis, or colon neoplasms. Abdominal pain, fever, distention, vomiting, night sweats, weight loss, and diarrhea are all indications of this localization [6]. Intestinal tuberculosis presenting with massive rectal bleeding is a rare manifestation accounting for only 5% of causes of lower gastrointestinal bleed [7]. To our knowledge, this is the first case report from Nepal. The diagnosis becomes more difficult in the absence of active pulmonary tuberculosis. The correct diagnosis is only 50% even in prevalent areas [8]. In undeveloped countries like Nepal, where the loss to follow-up is a big concern, delays in prompt diagnosis and treatment lead to severe morbidity and mortality [9].

The primary site of involvement was the ileocaecal area in our patient, and there were no clinical or radiological signs of pulmonary tuberculosis. The colonoscopy-guided biopsy is the investigation of choice in diagnosing ileocaecal and colonic tuberculosis. The acid-fast staining, culture, and gene XPERT have a sensitivity and specificity of (31%, 100%), (31%, 100%), and (95.7%, 100%) respectively [10]. Histological features of tuberculosis are caseous granulomas with conglomerate epithelioid histiocytes, giant cells with submucosal inflammation of predominantly lymphoplasmacytic type, while the microscopic features are lymphoid aggregates, pyloric metaplasia, dilated submucosal lymphatics, cryptitis, and crypt abscess [11]. However the classic histological features are seen only in 13–33% of patients with colonic tuberculosis [12]. Also, the rest show nonspecific findings. In our patient, histopathological findings did not show classical features of tuberculosis. In addition to caseous necrosis and acid-fast bacilli (which are found in only a small percentage of biopsy specimens from patients with intestinal tuberculosis), the size, number, and confluence of granulomas, the presence of ulcers lined by bands of epithelioid histiocytes, and disproportionate submucosal inflammation may help distinguish intestinal tuberculosis from Crohn's disease [12]. In our patient, there were multiple transverse ulcers suggestive of intestinal tuberculosis. Crohn's disease and intestinal tuberculosis can coexist rarely, and after successful treatment of tuberculosis, reevaluation is mandatory [13].

In a study in China, only 20% of patients were diagnosed with intestinal tuberculosis and the rest 80% were misdiagnosed [14]. Due to mimicking with other diseases such as Crohn's disease, a malignancy [3], misdiagnosis can lead to delay in treatment of other diseases as well as unnecessary drug toxicity from antitubercular therapy. Similarly, therapy with immunosuppressants in intestinal tuberculosis considering Crohn's disease can cause a flare up of tuberculosis and significant morbidity and mortality [8]. A single cost-effective investigation is still not available for intestinal tuberculosis, and the diagnosis reached on the therapeutic basis in 20% of cases [15].

The treatment of gastrointestinal tuberculosis is the same as that of pulmonary tuberculosis constituting a regimen of four drugs isoniazid, rifampicin, pyrazinamide, and ethambutol for initial 2 months followed by isoniazid and rifampicin for 4 months. Surgical treatment is instituted in intestinal obstruction, perforation, and fistulization refractory to ATT. The advancement of colonoscopy and gene XPERT has caused a decline in laparotomies.

Upper and lower gastrointestinal bleeding, fistulas at different sites, obstruction of the gut lumen, stricture formation, intussusception, perforation, anemia, malnutrition, malabsorption, weight loss, deficiency of essential vitamins and minerals, and chronic inflammatory demyelinating polyneuropathy have all been reported in patients with intestinal tuberculosis [13]. Surgical bypassing of concerned intestinal segments, radical excision of implicated segments, or conservative operations such as strictureplasty are used to treat intestinal tuberculosis [13].

Massive hematochezia can lead to rapid fall in the level of hemoglobin level as in our patient which can be managed with the transfusion of whole blood. For complications such as free perforation, substantial bleeding, total obstruction, abscess formation, big fistulas, and unresponsiveness to antimicrobial medications, surgery is the next best option after drug therapy. The most common complication is obstruction; individuals who have multiple and/or lengthy strictures are less likely to respond to medical treatment. Colonoscopic balloon dilation, which has been proved to be a viable option, can be used to treat easily accessible, short, and fibrous tuberculous ileal strictures that are causing subacute obstructive symptoms. Despite the lack of expertise, this approach looks safe and may eliminate the need for surgery. In our case, surgery was not opted for the treatment.

## 4. Conclusions

Although severe hematochezia is a rare symptom of intestinal tuberculosis, it should be considered a differential diagnosis in patients who come with rectal bleeding in tuberculosis-endemic areas. Physicians should be aware of the misdiagnosis due to the presentation of rectal bleeding that can lead to the potential complications.

## Data Availability

All the required information is in the manuscript itself.
